# Efficacy of combining oral Chinese herbal medicine and NB-UVB in treating psoriasis vulgaris: a systematic review and meta-analysis

**DOI:** 10.1186/s13020-015-0060-y

**Published:** 2015-09-26

**Authors:** Lihong Yang, Claire Shuiqing Zhang, Brian May, Jingjie Yu, Xinfeng Guo, Anthony Lin Zhang, Charlie Changli Xue, Chuanjian Lu

**Affiliations:** Evidence Based Medicine and Clinical Research Service Group, Guangdong Provincial Hospital of Chinese Medicine, Guangzhou, China; Department of Dermatology, Guangdong Provincial Hospital of Chinese Medicine, Guangzhou, China; The 2nd Clinical College of Guangzhou University of Chinese Medicine, Guangzhou, China; Guangdong Provincial Academy of Chinese Medical Sciences, Guangzhou, 510120 China; China-Australia International Research Centre for Chinese Medicine, School of Health Sciences, RMIT Health Innovations Research Institute, RMIT University, PO Box 71, Bundoora, Melbourne, VIC 3083 Australia

## Abstract

**Background:**

The combination of a Chinese herbal medicine (CHM) bath and narrowband ultraviolet B (NB-UVB) improved the efficacy of NB-UVB treatment of psoriasis vulgaris, but bath therapy is inconvenient. Oral CHM plus NB-UVB has been tested in clinical practice. This study aims to evaluate whether adding oral CHM could be beneficial for NB-UVB therapy by a systematic review and meta-analysis.

**Methods:**

Nine English and Chinese databases were searched from their inception to April 2014. Randomized controlled trials (RCTs) comparing the combination of orally administered CHM and NB-UVB with that of CHM placebo and NB-UVB or NB-UVB alone for treating psoriasis vulgaris and reporting Psoriasis Area Severity Index (PASI) outcomes were included. A systematic review, meta-analysis, risk of bias assessment and the GRADE assessment were conducted in accordance with Cochrane Collaboration methodology to assess the evidence for efficacy outcome. Data were analyzed in RevMan5.2.

**Results:**

Eighteen eligible RCTs (n = 1416) were included for systematic review, and 17 (n = 1342) of them were included in meta-analysis. Risk of bias in terms of blinding was high and so was in publication bias. Quality of evidence was low according the GRADE assessment. PASI-60 or above [risk ratio (RR) = 1.35, 95 % confidence interval (CI) 1.26–1.45, I^2^ = 5 %, number needed to treat = 4.27] and PASI-90 or above (RR = 1.71, 95 % CI 1.45–2.01, I^2^ = 0 %, number needed to treat = 5.92) were higher in the intervention group. The combination treatment conferred a 24 % benefit of PASI-60 or above (83 vs 59 %, RR = 1.35, 95 % CI 1.26–1.45, *P* < 0.01). The incidence of NB-UVB-induced adverse events was lower in the intervention group (95/464 vs 123/428, RR = 0.66, 95 % CI 0.46–0.96, *P* < 0.01). Mild gastrointestinal reactions (2.87 %) and liver function impairments (0.68 %) were reported in the intervention group. No serious adverse events were reported.

**Conclusion:**

The orally administrated CHM combined with NB-UVB in treating psoriasis vulgaris showed improved efficacy but quality of evidence was low.

**Electronic supplementary material:**

The online version of this article (doi:10.1186/s13020-015-0060-y) contains supplementary material, which is available to authorized users.

## Background

Psoriasis is a common, chronic and recurrent inflammatory skin disease with a global prevalence of 3–4 % [1]. The most common form, psoriasis vulgaris, affects 80–90 % of patients with psoriasis [2]. Ultraviolet-based therapies including psoralen plus ultraviolet A (PUVA), narrow-band ultraviolet B (NB-UVB) and broad-band ultraviolet B (BB-UVB) are effective treatments for psoriasis vulgaris. There is an increased risk of developing skin cancer following PUVA therapy [3, 4], although NB-UVB is more effective than BB-UVB and less effective than PUVA. Therefore, NB-UVB is preferred in the clinical management of psoriasis vulgaris [3, 5]. Because the clearance rate of NB-UVB treatment was reported in 51–75 % [6], adjuvant treatments are used to improve the effectiveness. Chinese herbal medicine (CHM) has been used for treating psoriasis vulgaris in China. The combination of a CHM bath and NB-UVB was beneficial in psoriasis vulgaris patients [7], but this approach has limitations in practice because a 30-min bath was required prior to each NB-UVB session. Therefore, the combination of oral CHM and NB-UVB has been used in clinical practice for treating psoriasis vulgaris [8]. However, it is still unclear if oral CHM combined with NB-UVB is beneficial for psoriasis vulgaris treatment.

This study aims to evaluate whether oral CHM could provide an added beneficial effect on NB-UVB therapy by a systematic review and meta-analysis.

## Methods

### Search strategies

Nine English and Chinese electronic databases, namely, the PubMed, Embase, CINAHL, CENTRAL, AMED, China National Knowledge Infrastructure (CNKI), Chinese BioMedical Literature (CBM), Chinese Science Journals Full Text (CQVIP) and Wanfang databases, were searched from their inceptions to April 2014. Search terms were grouped in three categories: (1) condition (psoriasis); (2) intervention (CHM, acupuncture, moxibustion, and other traditional Chinese medicine therapies); and (3) study type (randomized controlled trials), adjusted for each database (Table 1). The cited references of review articles were searched to identify additional studies.

**Table 1:** Search strategy in PubMed

Categories	Search terms
Condition	1. Skin diseases, papulosquamous (MeSH)
	2. Psoriasis (MeSH)
	3. Psoriases
	4. Pustulosis palmaris et plantaris
	5. Palmoplantaris pustulosis
	6. Pustular psoriasis of palms and soles
	7. Psoria^a^
	8. #1–#7/OR
Intervention	9. Medicine, Chinese traditional (MeSH)
	10. Chinese traditional medicine
	11. Chinese herbal drugs
	12. Chinese drugs, plant
	13. Medicine, traditional
	14. Ethnopharmacology
	15. Ethnomedicine
	16. Ethnobotany
	17. Medicine, Kampo
	18. Kampo
	19. TCM
	20. TCM
	21. Medicine, ayurvedic^b^
	22. Phytotherapy
	23. Herbology
	24. Plants, medicinal
	25. Plant preparations
	26. Plant extracts
	27. Plants, medicine
	28. Materia medica
	29. Single prescription
	30. Chinese medicine herb
	31. Herbal medicine
	32. Herbs
	33. #9–#32/OR
Study type	34. Randomized controlled trial (pt)
	35. Controlled clinical trial (pt)
	36. Randomized (tiab)
	37. Randomly (tiab)
	38. Placebo (tiab)
	39. Trial (tiab)
	40. Groups (tiab)
	41. #34–#40/OR
	42. #8 AND #33 AND #41

^a^ Any word starts from Psoria

^b^ Traditional Indian Herbal Medicine

### Study selection criteria

Randomized controlled trials (RCT) that compared orally administered CHM plus NB-UVB with CHM placebo plus NB-UVB or NB-UVB alone for psoriasis vulgaris were selected. The Psoriasis Area Severity Index (PASI) was used in clinical trials for outcome measurement following psoriasis treatment. The PASI measures the redness, thickness and scaling of the lesions and the area of involvement, with a total score ranging from 0 to 72. Reduction of disease severity was assessed based on the percent change in PASI score. PASI-75, referring to a 75 % reduction in PASI, was considered effective in psoriasis treatment [6]. Only studies reporting PASI scores as an outcome were included.

Studies were excluded if they met the following exclusion criteria: (1) participants had comorbidities; (2) CHM products included Western pharmaceuticals; and (3) there were co-interventions that used anti-psoriatic drugs or Chinese medicine treatments other than oral CHM.

### Data extraction and quality assessment

Two authors (LY, CZ) independently extracted and cross-checked data using a pre-defined sheet including: study location, setting, participants, sample size, treatment duration, intervention details, dropouts, outcome measures and adverse events (AE). If data were incomplete or appeared incorrect, attempts were made to contact the authors to request additional information or clarifying data. LY and CZ conducted risk of bias assessments independently using the Cochrane Collaboration tool for assessing risk of bias [9]. The grades of recommendation, assessment, development and evaluation (GRADE) approach was used to assess the quality of the evidence for individual outcomes, considering risk of bias, heterogeneity, directness of evidence, precision of effect estimation, and risk of publication bias. Disagreements were resolved by discussion with the two other authors (XG, AZ).

### Data analysis

Meta-analysis was performed in RevMan5.2. Risk ratios (RR) with 95 % confidence intervals (CI) for dichotomous data and mean differences (MD) with 95 % CIs for continuous data were reported. Data were pooled to estimate the effect size by the random-effects model [9]. Subgroup analysis was performed according to treatment duration, baseline severity, the CHM formulas and main formula ingredients. Sensitivity analysis was performed taking account of the risk of bias to assess the reliability of the pooled results. Dropouts or withdrawals were considered ineffective cases in an intention-to-treat (ITT) analysis to obtain a conservative estimation. Publication bias was assessed by funnel plots and Egger’s linear regression test. An asymmetrical funnel plot or *P* < 0.05 in Egger’s test indicated a publication bias.

## Results

**Fig. 1:**
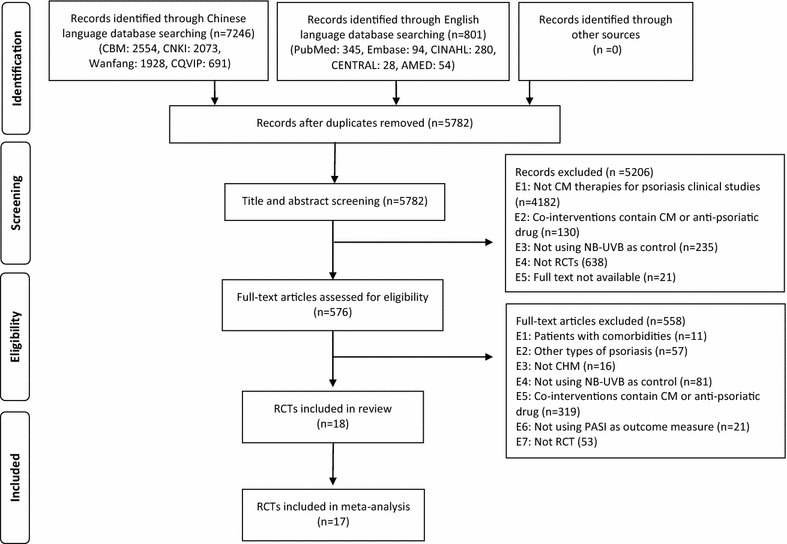
PRISMA flow chart of the study selection process

The initial searches yielded 5782 articles. After removing duplicates and screening titles and abstracts, 576 full-text articles were retrieved for further judgment. Eighteen articles were included [8, 10–26]. The study selection process is presented in Fig. 1.

### Description of the included studies

The 18 included trials, involving 1416 participants with psoriasis vulgaris, were conducted in hospitals in China and published in Chinese from 2004 to 2014. Characteristics of the included studies are summarized in Table 2. All included studies evaluated oral CHM added to NB-UVB vs NB-UVB alone, and six [8, 13, 14, 18, 19, 22, 24] were three-arm trials that included a comparison with oral CHM alone (not analyzed in this study). A placebo was not used in any included study. Two studies used disease severity as one of the inclusion criteria. Fu et al. [11] recruited psoriasis vulgaris participants with an affected body surface area (BSA) >10 % and Sheng et al. [19] recruited psoriasis vulgaris participants with an affected BSA >10 % and PASI score ≥10. Ten studies reported mean PASI score at baseline. The mean PASI scores were <10 in two studies [10, 17], >20 in one study [16], and ranged from 14.32 to 19.19 in the remaining seven studies [8, 14, 15, 18, 19, 22, 26]. Eight studies recruited psoriasis vulgaris participants targeting specific Chinese medicine patterns [8, 10, 15, 16, 21, 22, 25, 26].

**Table 2:** Characteristics, interventions and adverse events of the included studies

Author; year	No. of participants; dropouts (T/C)	Treatment; follow-up duration	CM pattern	Treatment interventions and ingredients	NB-UVB prescription (initial dosage; increment regimen; maximum dosage; frequency; total treatment session)	Adverse events (no. of participant reported AE)
Chen; 2010	46/44; 0/0	2; 6 months	BS	*Hua yu xiao yin tang*: *dang gui* 21 g, *dan shen* 21 g, *chi shao* 30 g, *tao ren* 12 g, *hong hua* 12 g, *hong teng* 15 g, *tu fu ling* 30 g, *bai hua she she cao* 21 g, *she tui* 15 g, *jin yin hua* 21 g, *ban lan gen* 21 g, *huai hua* 15 g, *bai xian pi* 30 g, *gan cao* 9 g	NB-UVB: 0.3; 0.2; 3 J/cm^2^; 3/week reduce to 1/week; NS	T: erythema (1), pruritus (3), dry skin (2); C: erythema (3), pruritus (4), dry skin (5), burning sensation (2), skin aging (1). AE rate: 13 vs 34 % (*P* > 0.05)
Cui; 2005	(3-Arms) 20/20/20; 0/0/0	8 weeks; NS	① BH	1. *Liang xue huo xue tang*: *huai hua,* *bai mao gen,* *sheng di huang,* *zi cao*, *chi shao*, *dan shen*	NB-UVB (311 nm): 0.3; 0.1 J/cm^2^; NS; 2/week; 16 session	T: erythema (2), pruritus and dry skin (2); C: erythema (5), blister (1), pruritus and dry skin (4). AE rate: 12.5 vs 31.3 % (*P* < 0.05)
				2. *Yang xue jie du tang*: *ji xue teng, dang gui, dan shen, tian dong, mai dong, sheng di huang, tu fu ling, feng fang*		
				3. *Huo xue san yu tang: san leng, e zhu, tao ren, hong hua, ji xue teng, gui jian yu, bai hua she she cao*		
			② BD			
			③ BS			
Fu; 2007	68/56; 0/0	8 weeks; 1 year	NS	*Yang xue huo xue tang*: *shu di huang* 30 g, *e zhu* 18 g, *dang gui* 15 g, *he shou wu* 24 g, *xuan shen* 18 g, *san leng* 9 g, *dan shen* 24 g, *wu shao she* 15 g, *sheng huang qi* 24 g, *yi yi ren* 18 g, *shan yao* 15 g, *jin yin hua* 15 g, *gan cao* 6 g	NB-UVB (311 nm): 0.05; 0.1 J/cm^2^; NS; qod; 28 sessions	Erythema, dry skin, burning sensation and hyperpigmentation occurred in most of participants after phototherapy in both groups
Huang; 2006	38/42; 0/0	4 weeks; NS	NS	*Huo ba hua gen pian*: extract of *Kun ming shan hai tang*	NB-UVB (311 nm): 0.3;0.2; 2.5 J/cm^2^, 3/week; 12 sessions	T: erythema, pruritus and dry skin (22), mild nausea (3), transient ALT elevated (1);C: erythema, pruritus and dry skin (16). AE rate: 60 vs 42 % (*P* > 0.05)
Liu; 2011	62/60; 0/0	8 weeks; NS	NS	*Xiao yin ke li*: *sheng di huang*, *mu dan pi*, *shao yao*, *dang gui*, *ku shen*, *xuan shen*, *bai xian pi*, *hong hua*, f*ang feng*, *jin yin hua*, *niu bang zi*	NB-UVB (311 nm): 0.3; 0.1 J/cm^2^ or 15–20 %; 3 J/cm^2^; qod; 28 sessions	T: burning sensation at 1st treatment (9), pruritus (8), ALT or AST mild elevated (5), gastrointestinal upset (3); C: burning sensation at 1st treatment (8), pruritus (7). AE rate: 40 vs 25 % (*P* > 0.05)
Liu; 2013	(3-Arms) 48/48/48; 6/6/0	12 weeks; NS	NS	*Tui yin tang*: *sheng di huang* 30 g, *tu fu ling* 30 g, *dang gui* 15 g, *he shou wu* 15 g, *bai ji li*15 g, *nv zhen zi* 10 g, *huang jing* 10 g, *mai dong* 10 g, *wu shao she* 10 g, *wu gong* 10 g, *jin yin hua* 10 g, *mu dan pi* 10 g, *tao ren* 10 g, *hong hua* 10 g, *gan cao* 5 g	NB-UVB (311 nm): 0.5 J/cm^2^; 10–20 %; 2/week; 24 sessions	T: painful erythema (2), pruritus (2), abdominal distension (1); C: painful erythema (3), pruritus (2). AE rate: 11.9 vs 11.9 %
Lu; 2009	36/26; 0/0	4 weeks; 3 months	BD	Unnamed CHM decoction: *dang gui* 10 g; *sheng di huang* 30 g; *tian dong* 15 g; *mai dong* 15 g; *dan shen* 15 g; *ji xue teng* 30 g; *tu fu ling* 30 g; *bai zhu* 10 g; *bai xian pi* 15 g	NB-UVB: 0.2; 0.1 J/cm^2^; NS; 3/week; 12 sessions	T: pruritus and dry skin (7), hyperpigmentation (3), mild nausea and vomit (2); C: pruritus and dry skin (4), hyperpigmentation (2). AE rate: 33 vs 23 % (*P* > 0.05)
Lv; 2009	36/32; 0/0	8 weeks; NS	NS	*Ke yin wan*: *tu fu ling*, *quan shen*, *bai xian pi*, *bei dou gen*, etc.	NB-UVB (311–315 nm): 0.4–0.5 J/cm^2^; 10–20 %; 2.5 J/cm^2^; 2/week; 16 sessions	T: erythema and burning sensation (4), transient ALT or AST elevated (3); C: erythema and burning sensation (8). AE rate: 19 vs 25 % (*P* > 0.05)
Lv; 2010	43/41; 0/0	6 weeks; NS	WH	*Liang xue tong luo tang*: *shui niu jiao* 10 g, *dan shen* 20 g, *chi shao* 15 g, *mu dan pi* 15 g, *jiang can* 15 g, *di long* 10 g, *wu shao she* 15 g	NB-UVB (311 nm): according to skin type 5–0.7; 0.1; 1.6 J/cm^2^; qod; 21 sessions	T: diarrhea (3), erythema (3), pruritus or skin pigmentation (4); C: blister (1), erythema (5), pruritus or skin pigmentation (9); AE rate: 23 vs 37 % (*P* > 0.05)
Niu; 2012	(3-Arms) 35/35/35; 0/0/0	8 weeks; 6 months	NS	*Liang xue xiao feng tang*: *sheng di huang* 30 g, *xuan shen* 9 g; *bai shao* 12 g; *sheng shi gao* 30 g; *zhi mu* 9 g; *bai mao gen* 30 g; *niu bang zi* 9 g; *jing jie* 9 g; *fang feng* 9 g; *gan cao* 6 g; *sheng ma* 3 g; *jin yin hua* 15 g; etc.	NB-UVB: 0.2–0.5 J/cm^2^; 10–20 %; NS; 2/week; 16 sessions	Painful erythema (6)
Sheng; 2014	(3-Arms) 30/30/30; 0/0/0	12 weeks; NS	NS	*Tui yin tang*: *sheng di huang* 30 g, *tu fu ling* 30 g, *dang gui* 15 g, *he shou wu* 15 g, *bai ji li*15 g, *nv zhen zi* 10 g, *huang jing* 10 g, *mai dong* 10 g, *wu shao she* 10 g, *wu gong* 10 g, *jin yin hua* 10 g, *mu dan pi* 10 g, *tao ren* 10 g, *hong hua* 10 g, *gan cao* 5 g	NB-UVB: MED 0.5 J/cm^2^; 10–20 %; NS; 2/week; 24 sessions	T: mild burning sensation, pruritus or gastrointestinal reaction (4); C: mild burning sensation, pruritus (4). AE rate: 13.3 vs 13.3 %
Sun; 2010	32/20; 0/0	NS; NS	NS	*Hua yu bai bi jiao nang*: *sheng di huang*, *mai dong*, *tian dong*, *dang gui*, *he shou wu*, *xuan shen*, *huai hua*, *she chuang zi*	NB-UVB: 0.35 J/cm^2^; 15 %; NS; NS; 29 sessions	T: pruritus (10, 31 %), dry skin (8, 25 %); C: pruritus (13, 55 %), dry skin (16, 80 %)
Wang; 2006	32/32; 0/0	1; 1 month	NS	*Liang xue huo xue fu fang*: *da qing ye* 15 g, *sheng di huang* 30 g, *huang qin* 12 g, *zi cao* 9 g, *dan shen* 12 g, *chi shao* 6 g, *mu dan pi* 9 g, *dang gui* 12 g, *tu fu ling* 30 g, *bai xian pi* 9 g, *jing jie* 6 g	NB-UVB: 0.4; 0.1 J/cm^2^; NS; qod, 12-16 sessions	T: erythema (2), pruritus and dry skin (2); C: blister (1), erythema (5), pruritus and dry skin (4); AE rate: 12.5 vs 31 % (*P* > 0.05)
Wang; 2008	(3-Arms) 53/53/53; 0/0/0	1; 6 months	BH	*Shen di chong ji*: *sheng di huang* 15 g, *xuan shen* 12 g, *mu dan pi* 12 g, *chi shao* 12 g, *dan shen* 15 g, *ban lan gen* 15 g, *tu fu ling* 15 g, *wu shao she* 10 g, *chan tui* 10 g, etc.	NB-UVB: 0.4–0.4 J/cm^2^; 15–25 %; 2.9 J/cm^2^; qod; 10–15 sessions	Erythema, pruritus, dry skin and hyperpigmentation occurred in most of participants after phototherapy in both groups
Xia; 2011	40/40; 0/0	2 months; 0	BH	*Liang xue xiao feng tang*: *sheng di huang* 30 g, *sheng shi gao* 30 g, *bai shao* 12 g, *bai mao gen* 30 g, *jin yin hua* 15 g, *jing jie* 9 g, *fang feng* 9 g, *xuan shen* 9 g, *zhi mu* 9 g, *niu bang zi* 9 g, *gan cao* 6 g, *sheng ma* 3 g	NB-UVB (311–313 nm): 0.3; 0.1 J/cm^2^; NS; 2/week; 16 sessions	Skin redness and itch (5)
Yan; 2011	(3-Arms) 30/30/30; 0/1/0	8 weeks; 0	NS	*Wu she jie du wan*: *wu shao she*, *wu gong*, *jiang can*, *she tui*, *niu bang zi*, *ku shen*, *dang gui*, *sheng di huang*, *mai dong*, *he shou wu*, *dan shen,* *e zhu*, *huang qin*, *gan cao*	NB-UVB: 0.35 J/cm^2^; 15 %; NS; qod; 29 sessions	Pruritus and dry skin (17), painless erythema (3)
Yu; 2004	37/37; 0/0	8 weeks; 1 year	① BD	1. *Xue zao fang*: d*ang gui* 20 g, *mai dong* 10 g, *xuan shen* 10 g, *tu fu ling* 30 g, *bai hua she she cao* 30 g	NB-UVB: according to skin type 0.03–0.05 J/cm^2^; 30–50 %; NS; 3/week reduce to 1/week; NS	T: skin redness and itch (5); C: skin redness and itch (3); AE rate: 13 vs 8 % (*P* > 0.05)
				2. *Huo xue fang*: *zi cao* 15 g, *chi shao* 20 g, *mu dan pi* 20 g, *dan shen* 20 g, *di long* 20 g, *ji xue teng* 20 g, *yi mu cao* 20 g		
			② BS			
Zhong; 2007	42/42; 0/0	2 months; 0	BD	*Xiao yin ke li*: *sheng di huang*, *dang gui*, *chi shao*, *chuan xiong*, *bai hua she she cao*, *zi cao*, *tu fu ling*, *e zhu*, *wu mei*, *gan cao*. etc.	NB-UVB (311 nm): 50 %MED 0.4; 0.05–0.1; 2.2 J/cm^2^; 3/week; 24 sessions	T: Nausea, loose stools at the beginning of treatment (12)

All studies provided NB-UVB for all participants in both treatment group and control group

*T* treatment group, *C* control group, *NS* not-stated, *CM* Chinese medicine, *NS* not-stated, *BH* blood heat, *BS* blood stasis, *BD* blood dryness, *WH* wind heat, *g* *gram*, *NB-UVB* narrow-band ultraviolet B, *MED* minimal erythema dose, *qod* every 2 days, *ALT* alanine aminotransferase, *AST* anti-aspartate aminotransferase

### Interventions

All studies reported that NB-UVB phototherapy was consistently prescribed to intervention groups and control groups. However, there was variation across studies in terms of the initial dosage, dosage increment regimen, treatment frequency and total number of treatment sessions. Most of the studies applied phototherapy two to three times per week for 10–29 treatment sessions in total.

Huang et al. [12] used a single herb extract product; the other 17 studies used multi-ingredient formulas in decoctions [10, 11, 13, 16, 18, 19, 21, 25], pills [12, 17, 24], capsules [20], or granules [14, 22, 26]. CHM was administered daily. No two studies used an identical herbal formula. Although all formulas varied across studies (Table 2), there were similarities in the herb ingredients used. The most frequently used herbs were *Rehmannia glutinosa* (*sheng di huang*) in 13 studies [8, 11, 13–15, 18–24, 26], *Angelica sinensis* (*dang gui*) in 11 studies [10, 11, 13–15, 19, 20, 23, 24, 26], *Smilax glabra* (*tu fu ling*) in nine studies [8, 10, 13, 15, 17, 19, 22, 23, 26], *Salvia miltiorrhiza* (*dan shen*) in eight studies [10, 11, 15, 16, 22–24], and *Glycyrrhiza uralensis* (*gan cao*) in eight studies [10, 11, 13, 18, 19, 21, 24, 26]. Furthermore, three herbs, *R. glutinosa*, *A. sinensis*, and *S. glabra*, were used together in six studies [8, 13, 15, 19, 23, 26]. The details of formula and herbs used by included studies can be found in Additional file 1: Table S1.

### Treatment and follow-up durations

Sun et al. [20] only reported the total number of phototherapy treatment sessions. The treatment durations of the other 17 studies ranged from 4 to 12 weeks, and ten studies were of 8 weeks (2 months).

Seven studies included follow up with participants after treatment. Four studies followed all included participants for 3 months [15], 6 months [22, 23], and 1 year [25]. Three studies followed participants who achieved PASI-90 for 6 months [10, 18] and 1 year [15].

### Outcome measures

Seventeen of the 18 studies reported the numbers of participants who achieved PASI-95 or PASI-90, and PASI-60 (at least 95, 90, 60 % reduction in PASI), with reference to the Consensus of Diagnosis and Treatment of Psoriasis Vulgaris with Integrative Medicine [27]. One study reported PASI-95 and PASI-70 [16]. As PASI-50 was taken as the minimum clinically meaningful improvement [6, 28], PASI-60 and PASI-70 were conservatively pooled as PASI-60 to analyze the effect of the combination of oral CHM and NB-UVB. Four studies reported the relapse rates at the end of the follow-up [10, 11, 18, 23], but the definitions of relapse were not stated.

### Dropouts and withdrawals

Liu and Sheng [13] reported that one participant in the treatment group and two in the control group were excluded after randomization because of comorbidities, and there were five dropouts in the treatment group and four dropouts in the control group for low compliance. Yan et al. [24] reported one withdrawal in the NB-UVB group owing to AEs, but a phototoxicity reaction was not observed. No dropout was reported in the other 16 studies.

### Overall efficacy

One study was not included in the meta-analysis, because the outcome measures were PASI-25 and PASI-10 [25]. This study reported that the effect rate was much higher in the combination group than in the NB-UVB group in terms of PASI-25 (78.4 vs 8.1 %). The results of the remaining 17 studies were pooled to estimate the efficacy of the combination treatment.

#### PASI-60 and above

**Fig. 2:**
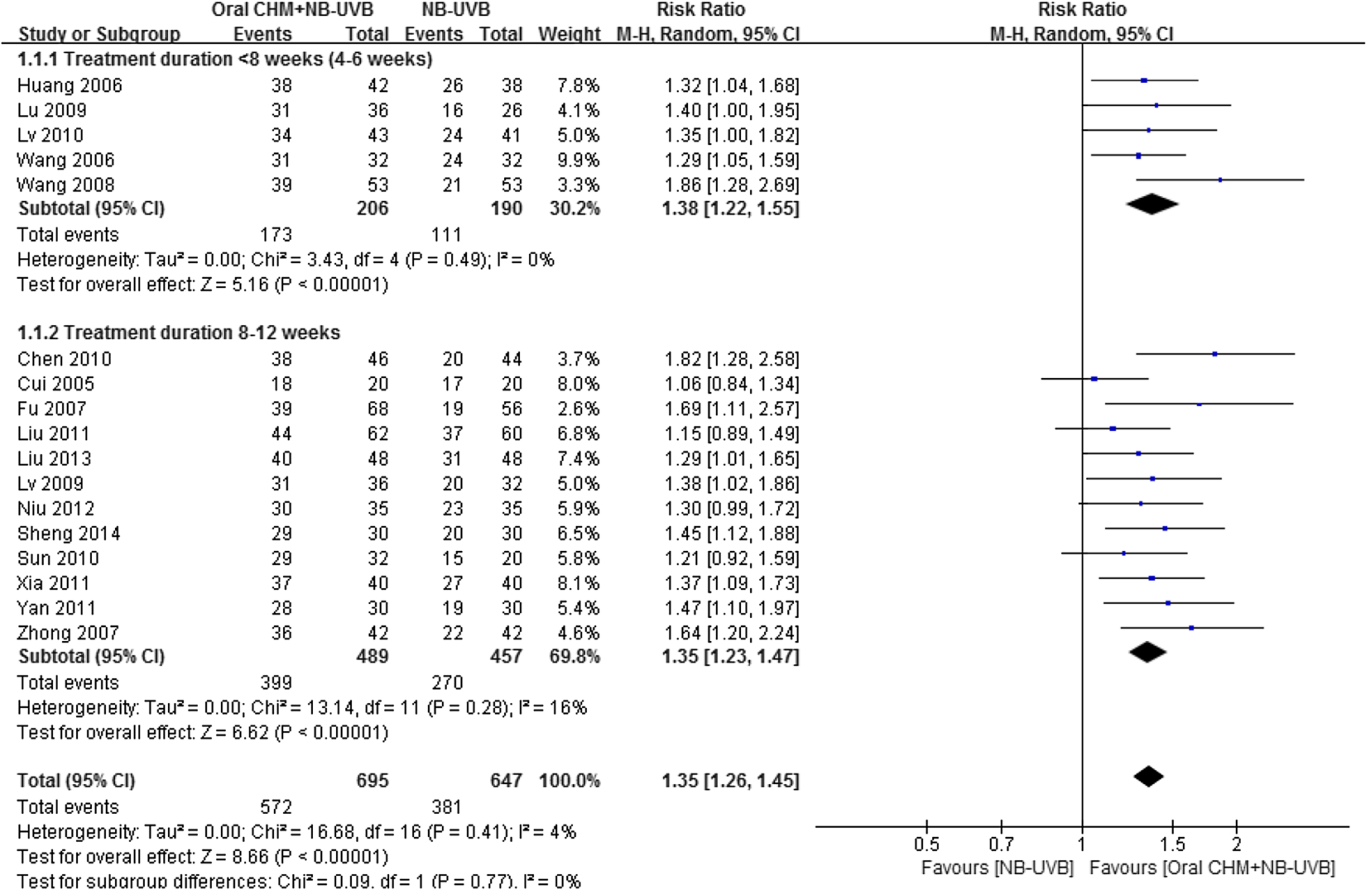
*Forest plot* of PASI 60

The mean percentage of participants who achieved PASI-60 at the end of treatment was 83 % in the combination group and 59 % in the NB-UVB group. PASI-60 was significantly higher in the combination group than in the NB-UVB group [Fig. 2, 17 studies, RR = 1.35, 95 % CI 1.26–1.45, *P* < 0.01, I^2^ = 5 %; number needed to treat (NNT) = 4.27].

The most common treatment duration of the included studies was 2 months. Subgroup analysis showed that the pooled results were consistent between studies with 4–6 weeks treatment (five studies, RR = 1.38, 95 % CI 1.22–1.55, *P* < 0.01, I^2^ = 0 %) and studies with 8–12 weeks treatment (12 studies, RR = 1.34, 95 % CI 1.23–1.47, *P* < 0.01, I^2^ = 16 %). For the seven studies that included participants with moderate to severe psoriasis, the result for PASI-60 was similar to that for the total pool (RR 1.35, 95 % CI 1.17–1.56, *P* < 0.01, I^2^ = 45 %).

The most common herb combination used was *R. glutinosa*, *A. sinensis* and *S. glabra*. When the studies using this herb combination were pooled, the result was consistent with the result of the total pool (Table 3), but with moderate heterogeneity (six studies, RR = 1.31, 95 % CI 1.16–1.54, *P* < 0.01, I^2^ = 22 %). Of these six studies, one study [8] prescribed three different formulas targeting three different Chinese medicine patterns, and the above three-herb combination was only included in one of the formulas. However, PASI-60 was not reported separately according to the different formulas. Therefore, the data reported did not represent the outcome of the particular formula which contained the three common herbs. When this study was excluded, the result showed a superior effect for the combination treatment without heterogeneity (RR = 1.37, 95 % CI 1.22–1.54, *P* < 0.01, I^2^ = 0 %).

**Table 3:** Results of meta-analysis

Outcomes	RR (95 % CI), I^2^, NNT
PASI-60 or above	
17 studies	1.35 (1.26, 1.45), I^2^ = 5 %, 4.27
Studies with <8 (4–6) weeks treatment (five studies)	1.38 (1.22, 1.55), I^2^ = 0 %
Studies with 8–12 weeks treatment (12 studies)	1.34 (1.23, 1.47), I^2^ = 16 %
Studies with moderate to severe psoriasis (baseline PASI 14-20) (seven studies)	1.35 (1.17, 1.56), I^2^ = 45 %
Studies using three common herbs (di huang, dang gui, tu fu ling) (six studies)	1.31 (1.16, 1.47), I^2^ = 22 %
Studies using three common herbs (five studies, one study that used three formulae was excluded)	1.37 (1.22, 1.54), I^2^ = 0 %
Studies with low risk on sequence generation (five studies)	1.39 (1.23, 1.58), I^2^ = 0 %
PASI-90 or above	
17 studies	1.71 (1.45, 2.01), I^2^ = 0 %, 5.92
NB-UVB-induced AEs	
12 studies	0.66 (0.46, 0.96), I^2^ = 53 %
Studies with <8 (4–6) weeks treatment (four studies)	0.77 (0.42, 1.43), I^2^ = 62 %
Studies with 8–12 weeks treatment (eight studies)	0.60 (0.38, 0.94), I^2^ = 46 %

*RR* RISK ratio, *NNT* number need to treat

For the five studies judged low risk on sequence generation, the result (RR = 1.39, 95 % CI 1.23–1.58, *P* < 0.01, I^2^ = 0 %) was consistent with that for the total pool.

#### PASI-90

**Fig. 3:**
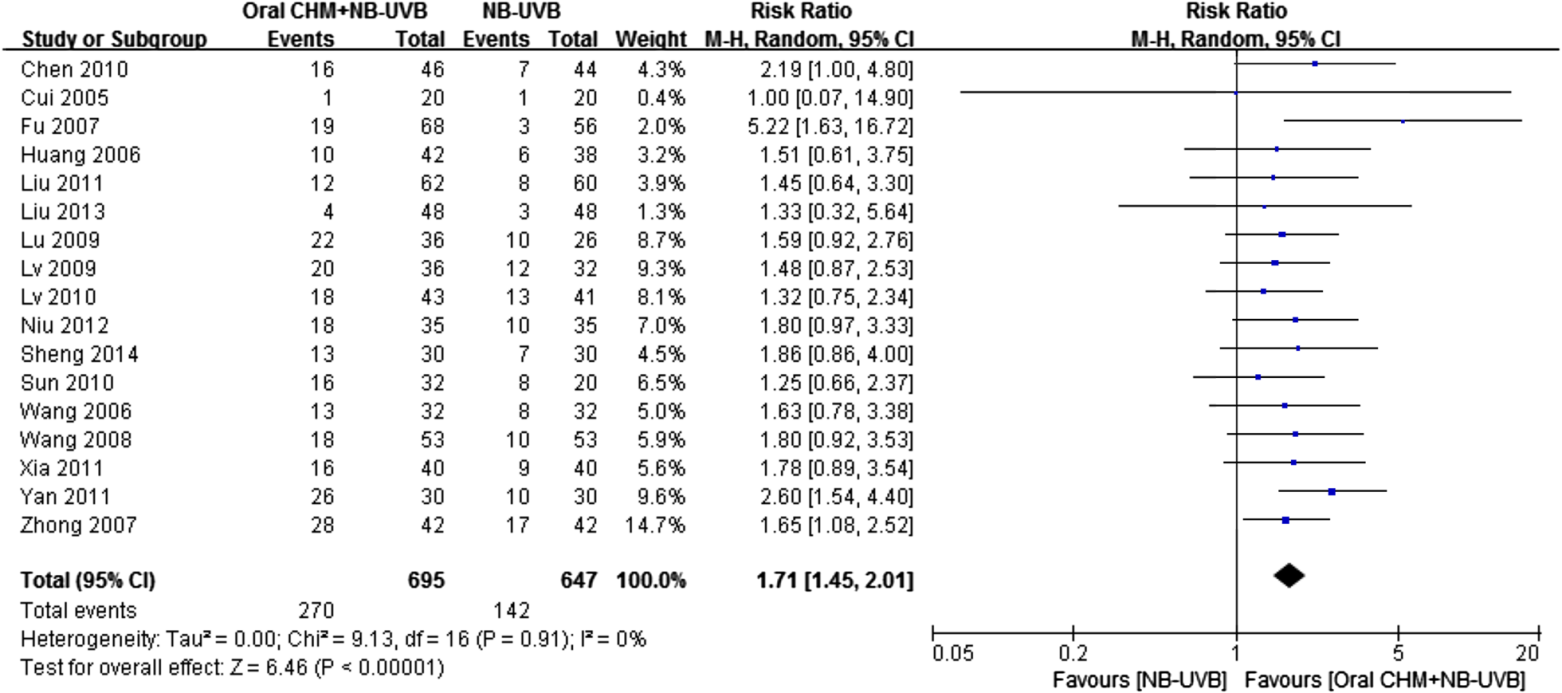
*Forest plot* of PASI 90

The meta-analysis of PASI-90 or above also favored the combination group (Fig. 3, 17 studies, RR = 1.71, 95 % CI 1.45–2.01, *P* < 0.01, I^2^ = 0 %; NNT = 5.92).

#### Post-follow-up relapse rate

The mean relapse rate of the three studies that followed participants who achieved PASI-90 was 11 % in the combination group and 60 % in the NB-UVB group 3 months–1 year after treatment. Because the definition of relapse was unclear and there were different follow up durations, meta-analysis of these studies was not performed.

### Adverse events

All studies reported AEs (Table 2). Zhong et al. [26] only reported AEs in the combination group, and three studies [18, 21, 24] reported skin reactions but did not mention which group these occurred in. One participant withdrew because of a phototherapy AE [24]; the other withdrawals were not associated with AEs. All AEs reported in the NB-UVB groups were skin reactions, with a mean rate of 31.58 % (8–80 %). AEs reported in the combination groups were skin reactions, gastrointestinal reactions and elevated liver enzymes. The total AE rate (24.42 %) in the combination groups was a little lower than that in NB-UVB groups. In addition, combination treatment lowered the incidence rate of skin reactions including pruritus, skin dryness, erythema, blistering and hyperpigmentation [95/464 (20.47 %) vs 123/428 (28.74 %); RR = 0.66, 95 % CI 0.46–0.96]. The subgroup analysis showed a significant difference in studies administering 8–12 weeks of treatment (eight studies, RR = 0.60, 95 % CI 0.38–0.94), but not in studies with a duration of 4–6 weeks (four studies, RR = 0.77, 95 % CI 0.42–1.43). Mild gastrointestinal reactions, such as nausea, vomiting, abdominal distension and stool change, were reported in the combination groups (21/732, 2.87 %) [12–14, 16, 17, 26]. Five cases of elevated liver enzymes were reported (5/732, 0.68 %). One case returned to normal within 1 month without additional treatment [12], three returned to normal by reducing the dosage of the CHMs [17], and the outcome was not reported for one [14]. The relationships between the AEs and the interventions were not further discussed in any study. No severe AE was reported.

### Risk of bias assessment

**Fig. 4:**
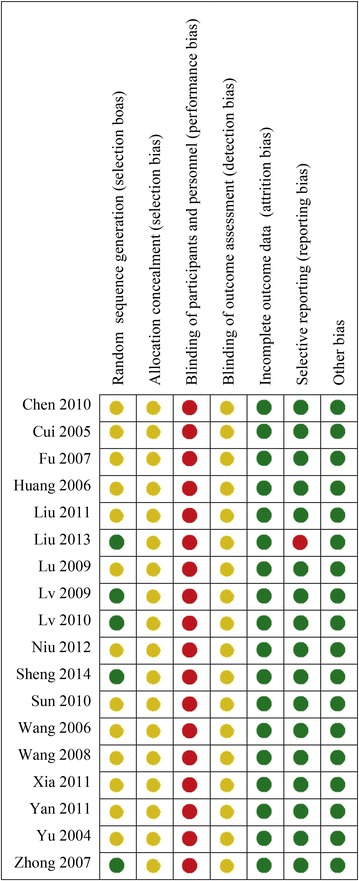
Risk of bias assessment results. *Green circle* for low risk of bias, *red circle* for high risk of bias, *yellow circle* for unclear risk of bias

Risk of bias assessments are presented in Fig. 4. Attempts were made to contact the original authors to obtain further information on 13 studies but the authors of 12 studies could not be reached and the author of one study refused to provide the further information we requested; therefore, risk of bias assessments were based on the published texts. For “sequence generation”, five studies that used random number tables (a table of random numbers is available in statistics textbooks or generated by computer) [14, 16, 17] and drawing lots (including coin-tossing, dice-throwing, or card-shuffling) [13, 19] were assessed as “low risk”, and the others were assessed as “unclear”. All studies were assessed as “unclear” for “allocation concealment” owing to the lack of information. All studies were assessed as “high risk” for “blinding of participants and research personnel”, because all studies used an add-on therapy and a placebo was not used for the CHM in any study. The blinding of outcome assessors was judged “unclear” because information was not provided. One study [24] had one withdrawal in the NB-UVB group, and it was assessed as “low risk” for “incomplete outcome data”, because the proportion of missing data was not enough to have an impact on the effect estimate. The amount of missing outcome data in one study [13] was 12.5 % and ITT analysis was not performed, so this study was assessed as “high risk” for “incomplete outcome data”. The others were assessed as “unclear”. All studies were “low risk” for “selective reporting” because all studies reported the outcomes pre-specified in the methods. All studies were assessed as “low risk” for “other bias”, which refers to the inappropriate influence of funders.

### Publication bias

**Fig. 5:**
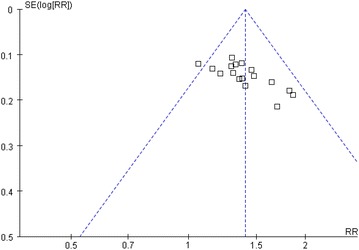
*Funnel plot* of the 17 studies that reported PASI-60

Asymmetrical funnel plots and significant Egger’s test (t = 3.88, *P* < 0.01) indicated potential publication bias in favor of more positive results for the smaller studies (Fig. 5).

### Quality of evidence

Owing to defects in study design and the risk of publication bias, the quality of evidence was downgraded to low according to the GRADE system (Table 4).

**Table 4:** Summary of findings

Oral CHM + NB-UVB compared to NB-UVB for psoriasis vulgaris					
Participant or population: participants with psoriasis vulgaris					
Settings: hospitals in China					
Intervention: oral CHM + NB-UVB					
Comparison: NB-UVB					

Outcomes	Illustrative comparative risks (95 % CI)		Relative effect (95 % CI)	No. of participants (studies)	Quality of the evidence (GRADE)
	Assumed risk	Corresponding risk			
	NB-UVB	Oral CHM + NB-UVB			
*PASI 60*	*Study population*		*RR 1.35* (1.26–1.45)	1339 (17 studies)	⊕ ⊕ ⊝⊝ *low* ^a,b^
Follow-up 4–12 weeks	*591 per 1000*	*797 per 1000* (744–857)			
	*Moderate*				
	*633 per 1000*	*855 per 1000* (798–918)			
*PASI 90*	*Study population*		*RR 1.71* (1.45–2.01)	1342 (17 studies)	⊕ ⊕ ⊝⊝ *low*
Follow-up 4–12 weeks	*219 per 1000*	*375 per 1000* (318–441)			
	*Moderate*				
	*233 per 1000*	*398 per 1000* (338–468)			

GRADE Working Group grades of evidence

High quality: further research is very unlikely to change our confidence in the estimate of effect

Moderate quality: further research is likely to have an important impact on our confidence in the estimate of effect and may change the estimate

Low quality: further research is very likely to have an important impact on our confidence in the estimate of effect and is likely to change the estimate

Very low quality: we are very uncertain about the estimate

^a^Blindness of participants and investigators is “high risk” in all studies

^b^Asymmetrical funnel plot and result of Egger’s test indicated potential publication bias

## Discussion

### Efficacy

The combination of oral CHM and NB-UVB was more effective than NB-UVB alone in achieving PASI-60 after 4–12 weeks of treatment (RR = 1.40, 95 % CI 1.30–1.50) with low heterogeneity (I^2^ = 4 %). The estimated NNT to have one achieve PASI-60 with 4–12 weeks treatment was around five. These results were consistent with a systematic review of CHM bath plus phototherapy for psoriasis vulgaris (RR = 1.25, 95 % CI 1.15–1.36) [7].

### Adverse events

Skin reactions such as pruritus and erythema were the most commonly reported AEs following combination treatment with oral CHM plus NB-UVB, and were the same as for NB-UVB phototherapy [8]. The combination treatment might lower incidence rates of NB-UVB-associated AEs after 8–12 weeks of treatment. Occasional mild gastrointestinal reactions (2.87 %) and rare liver function impairment (0.68 %) were reported in the combination group. All gastrointestinal reactions associated with oral CHM administration were transient and required no additional treatment. Liver function impairment presented as elevated liver enzymes. The studies did not discuss whether the impairment was caused by oral CHM administration, but three of the five participants returned to normal following a reduction in the dosage of CHMs, which suggested that the liver function impairment might be associated with the oral CHMs.

Overdose might be a factor leading to AEs. The dosages of ten herbs, *Polygonum multiflorum* (*he shou wu*), *R. glutinosa*, *S. miltiorrhiza*, *Dictamnus dasycarpus* (*bai xian pi*), *Curcuma zedoaria* (*e zhu*), *Sophora japonica* (*huai hua*), *Scolopendra* (*wu gong*), *Bombyx batryticatus* (*jiang chan*) and *Periostracum serpentis* (*she tui*), prescribed in the included studies, exceeded the dosages recommended in the China Pharmacopoeia 2010 [29]. The latest information bulletin on drug adverse reactions published by the China Food and Drug Administration reported that *P. multiflorum* and products containing this plant might be associated with liver function impairment [30], and that overdose and long-term usage could increase the risk of adverse reactions.

### Potential mechanisms of action of oral CHM combined with NB-UVB

#### Anti-psoriatic actions of NB-UVB

Suppression of epidermal hyperproliferation by inhibition of keratinocyte nuclear DNA synthesis is an important mechanism of action of UVB in the treatment of psoriasis [31]. UVB also has anti-inflammatory effects, with inhibition of T cell activation, induction of T cell apoptosis and reduction of the release of a variety of proinflammatory cytokines [6].

#### Anti-psoriatic actions of herbal medicines

Extracts, compounds or both, of all of the five herbs commonly used in multiple studies, exhibited anti-inflammatory effects [32]. In a guinea pig ear model of a psoriasis-like lesion induced by propranolol, parakeratosis pathological changes were significantly alleviated and epidermal proliferation was inhibited by catapol treatment (a major compound of *R. glutinosa*) [33], but an extract of *R. glutinosa* did not show an anti-proliferative effect on human epidermal keratinocytes (HaCaT cells) in vitro [34]. *S. miltiorrhiza* showed effects on inflammation, proliferation and angiogenesis [35]. In psoriasis-like lesions in guinea pig ears induced by topical propranolol, injection of angelica polysaccharide (a compound in *A. sinensis*) increased keratinocyte apoptosis [36] and decreased the expression level of proliferating cell nuclear antigen [37]. *S. glabra* exhibited anti-inflammatory effects and antitumor effects, and inhibited cellular immunity [38]. Anti-proliferative effects of *S. glabra* were observed in an in vivo study using a mouse vaginal epithelium model and a mouse tail model [39], but there was a negative result observed in an in vitro study using the HaCaT cell line [34]. Glycyrrhetinic acid (a compound in *G. uralensis*) had a dose-dependent anti-proliferative effect [40].

#### Synergetic interaction between NB-UVB and herbal medicine

Psoralen in combination with NB-UVB was more effective than NB-UVB alone [41]. Herbs with a high content of psoralen such as *Psoralea corylifolia* (*bu gu zhi*) [42] were not used in the included studies. *Saposhnikovia divaricata* (*fang feng*), which was used in three studies, contains psoralens such as 5-methoxypsoralen [43]. *A. sinensis*, which is used in 11 studies, and other *Angelica* species contain coumarins with photosensitising effects [44]. However, studies are required to examine the effects of combining coumarin-containing herbs and NB-UVB.

The combination treatment reduced the incidence of NB-UVB-induced AEs. Several herbal medicines showed protective effects against UVB-induced skin damage and can relieve pruritus. Survival of normal HaCaT cells pre-treated with a water extract of *S. miltiorrhiza* prior to UVB exposure was significantly higher than that of cells without pre-treatment [45]. In addition, *S. miltiorrhiza* showed anti-inflammatory effects by reducing secretion of TNF-α and IL-1β. Similar results were found in research on *Scutellaria baicalensis* (*huang qin*) and *Ligusticum chuanxiong* (*chuan xiong*) [46]. Licoflavone and glycyrrhizin (compounds from *G. uralensis*), exhibited photo-protective effects on HaCaT cells irradiated by UVB radiation [47], and UV protective effects for glycyrrhizin and extracts of *S. baicalensis* and *S. divaricata* were reported [48]. In a guinea pig foot pruritus model induced by histamine phosphate treatment, catapol (a compound in *R. glutinosa*) significantly relieved pruritus [33].

### Strengths and limitations of this meta-analysis

The 17 studies included in the meta-analysis were comparable in that they assessed a CHM combined with NB-UVB compared with NB-UVB treatment alone, with the difference in PASI score as an outcome. Although the meta-analysis results for the subgroups and sensitivity analyses tended to be consistent with those for the total pool, in terms of the quality of the evidence, a number of issues led to this being downgraded to low. Because none of the studies used a placebo for the CHM in the control arm, blinding of participants could not be achieved. Consequently, any placebo effect could not be distinguished from a genuine add-on biological therapeutic effect. Sequence generation was unclear in 13 studies; we are not sure whether these studies were truly randomized. The asymmetrical funnel plot showed that the smaller studies reported more positive results, suggesting that there might be a publication bias.

Other issues that may impact upon the interpretability of the results were variability in the severity of psoriasis in the participants, the duration and dosage of the NB-UVB and CHM treatments, and variability in the composition and quality of the CHMs. It was not feasible to explore all of these issues owing to limitations in the available data, but the sub-group analyses that were undertaken were consistent with the PASI-60 results for the total pool. Additionally, all included studies were conducted in China and the samples were restricted to Chinese populations, which might limit the generalization of the results. Adverse events were not reported during the follow-up periods and the criteria for relapse rate during follow up were not well defined. As such, the long-term efficacy of and the adverse events associated with the combination treatment could not be evaluated in this study.

### Implications for clinical practice and further research

The results of this study suggested that, in clinical psoriasis vulgaris treatment, the three most frequently used herbs, *R. glutinosa*, *A. sinensis*, *S. glabra*, could be considered the core herbs for treating participants undergoing NB-UVB treatment. The dose for each herb should be in strict compliance with the China Pharmacopoeia and regular monitoring of liver function is needed during treatment.

In further research, the disease severity of psoriasis participants should be clearly reported, and a stratified (by disease severity) analysis should be performed. A placebo for the CHM should be used in the control group to enable effective blinding. For alignment with international studies, PASI-50 and PASI-75 should be reported. Remission or relapse should be clearly defined and reported to evaluate long-term effectiveness. In addition, whether combination treatment could reduce the number of treatment sessions and/or the cumulative dose of NB-UVB should be investigated. Furthermore, a study on the long-term safety of this combination treatment is needed.

## Conclusion

Orally administered CHM combined with NB-UVB showed improved efficacy for treating psoriasis vulgaris but quality of evidence was low.

## Additional file

10.1186/s13020-015-0060-y Herbs used in the included studies.

## Abbreviations

CHM: Chinese herbal medicine
RCT: randomized controlled trial
PUVA: psoralen plus ultraviolet A
NB-UVB: narrowband ultraviolet B
BB-UVB: broad-band ultraviolet B
CNKI: China National Knowledge Infrastructure
CBM: Chinese BioMedical Literature
CQVIP: Chinese Science Journals Full Text
PASI: Psoriasis Area Severity Index
BSA: body surface area
AE: adverse event
GRADR: the grades of recommendation, assessment, development and evaluation
RR: risk ratio
MD: mean difference
CI: confidence interval
ITT: intention-to-treat
NNT: number needed to treat
HaCaT cell: human epidermal keratinocyte
TNF-α: tumor necrosis factor α
IL-1β: interleukin-1β
UV: ultraviolet
