# Supplementary material for: Efficacy of combining oral Chinese herbal medicine and NB-UVB in treating psoriasis vulgaris: a systematic review and meta-analysis
Source: Chin Med. 2015 Sep 26;10:27. doi: 10.1186/s13020-015-0060-y (PMC4583725; doi:10.1186/s13020-015-0060-y)
Supplement: Supplementary file 1 — 10.1186/s13020-015-0060-y Herbs used in the included studies. [file 13020_2015_60_MOESM1_ESM.docx]

Supplementary table 1 Herbs used in the included studies

| **Chinese name** | **Latin pharmaceutical name** | **Latin scientific name** |
| --- | --- | --- |
| *bai hua she she cao* (百花蛇舌草) | *Herba Hedyotis* | *Hedyotis diffusa* |
| *bai ji li* (白蒺藜) | *Fructus Tribuli* | *Tribulus terrestris* |
| *bai mao gen* (白茅根) | *Rhizoma Imperatae* | *Imperata cylindrica var. major* |
| *bai xian pi* (白鮮皮) | *Cortex Dictamni* | *Dictamnus dasycarpus* |
| *bai zhu* (白朮) | *Rhizoma Atractylodis Macrocephalae* | *Atractylodes macrocephala* |
| *ban lan gen* (板藍根) | [*Radix*](javascript:void(0);) [*Isatidis*](javascript:void(0);) | *Isatis indigotica* |
| *bei dou gen* (北豆根) | *Rhizoma Menispermi* | *Menispermum dauricum* |
| *chan tui* (蟬蛻) | *Periostracum Cicadae* | *Cryptotympana* species |
| *chi shao* (赤芍) | *Radix Paeoniae Rubra* | *Paeonia lactiflora, P. veitchii* |
| *chuan xiong* (川芎) | *Rhizoma Ligustici Chuanxiong* | *Ligusticum chuanxiong* |
| *da qing ye* (大青葉) | [*Folium*](javascript:void(0);) [*Isatidis*](javascript:void(0);) | *Isatis indigotica* |
| *dan shen* (丹參) | *Radix Salviae Miltiorrhizae* | *Salvia miltiorrhiza* |
| *dang gui* (當歸) | *Radix Angelicae Sinensis* | *Angelica sinensis* |
| *di long* (地龍) | *Lumbricus* | *Pheretima* species |
| *e zhu* (莪朮) | *Rhizoma curcumae* | *Curcuma zedoaria* |
| *fang feng* (防風) | *Radix Saposhnikoviae* | *Saposhnikovia divaricata* |
| *gan cao* (甘草) | *Radix Glycyrrhizae* | *Glycyrrhiza uralensis* |
| *gui jian yu* (鬼箭羽) | *-* | *Euonymus alatus* |
| *he shou wu* (何首烏) | *Radix Polygoni Multiflori* | *Polygonum multiflorum* |
| *hong hua* (紅花) | *Flos Carthami* | *Carthamus tinctorius* |
| *huai hua* (槐花) | *Flos Sophorae* | *Sophora japonica* |
| *huang jing* (黃精) | *Rhizoma Polygonati* | *Polygonatum sibiricum* |
| *huang qi* (黃芪) | *Radix Astragali seu Hedysari* | *Astragalus membranaceus* |
| *huang qin* (黃芩) | *Radix Scutellariae* | *Scutellaria baicalensis* |
| *ji xue teng* (雞血藤) | *Caulis Spatholobi* | *Spatholobus suberectus* |
| *jiang can* (僵蠶) | *Bombyx Batryticatus* | *Bombyx mori & Beauveria bassiana* |
| *jin yin hua* (金銀花) | *Flos Lonicerae* | *Lonicera japonica* |
| *jing jie* (荊芥) | *Herba Schizonepetae* | *Schizonepeta tenuifolia* |
| *Kun ming shan hai tang* (昆明山海棠) | *-* | *Tripterygium hypoglaucum* |
| *ku shen* (苦參) | *Radix Sophorae Flavescentis* | *Sophora flavescens* |
| *mai dong* (麥冬) | *Radix Ophiopogonis* | *Ophiopogon japonicus* |
| *mu dan pi* (牡丹皮) | *Cortex Moutan Radicis* | *Paeonia moutan* |
| *niu bang zi* (牛蒡子) | *Fructus Arctii* | *Arctium lappa* |
| *nv zhen zi* (女貞子) | *Fructus Ligustri Lucidi* | *Ligustrum lucidum* |
| *quan shen* (拳參) | *Rhizoma Bistortae* | *Polygonum bistorta* |
| *san leng* (三棱) | *Rhizoma Sparganii* | *Sparganium stoloniferum* |
| *shan yao* (山藥) | *Rhizoma Dioscoreae* | *Piper hancei* |
| *she chuang zi* (蛇床子) | *Fructus Cnidii* | *Cnidium monnieri* |
| *she tui* (蛇蛻) | *Periostracum Serpentis* | *Elaphe or Zaocys* species |
| *sheng di huang* (生地黃) | *Radix Rehmanniae Recens* | *Rehmannia glutinosa* |
| *sheng ma* (升麻) | *Rhizoma Cimicifugae* | *Cimicifuga foetida* |
| *shu di huang* (熟地黃) | *Radix Rehmanniae Preparata* | *Rehmannia glutinosa* |
| *shi gao* (石膏) | *Gypsum Fibrosum* | *Calcium sulfate dihydrate* |
| *shui niu jiao* (水牛角) | *Cornu Bubali* | *Bubalus bubalis* |
| *tao ren* (桃仁) | *Semen Persicae* | *Prunus persica* |
| *tian dong* (天冬) | *Radix Asparagi* | *Asparagus cochinchinensis* |
| *tu fu ling* (土茯苓) | *Rhizoma Smilacis Glabrae* | *Smilax glabra* |
| *wu gong* (蜈蚣) | *Scolopendra* | *Scolopendra subspinipes mutilans* |
| *wu mei* (烏梅) | *Fructus Mume* | *Prunus mume* |
| *wu shao she* (烏梢蛇) | *Zaocys* | *Zaocys dhumnades* |
| *xuan shen* (玄參) | *Radix Scrophulariae* | *Scrophularia ningpoensis* |
| *yi mu cao* (益母草) | *Herba Leonuri* | *Leonurus heterophyllus* |
| *yi yi ren* (薏苡仁) | *Semen Coicis* | *Coix lacryma-jobi* |
| *zhi mu* (知母) | *Rhizoma Anemarrhenae* | *Anemarrhena asphodeloides* |
| *zi cao* (紫草) | *Radix Lithospermi* | *Lithospermum erythrorhizon* |
